# Biomechanical analysis of stair walking patterns and their implications for knee health in young males

**DOI:** 10.3389/fpubh.2025.1725056

**Published:** 2026-01-05

**Authors:** Yang Sun, Qingkang Mei, Danyang Kou, Jiujiang Liu, Yi Yang, Lian Duan, Yuan Gao

**Affiliations:** 1 School of Physical Education, Yanshan University, Qinhuangdao, China; 2 Department of Physical Education, Hebei Institute of Mechanical and Electrical Technology, Xingtai, China; 3 China Volleyball College, Beijing Sport University, Beijing, China; 4 Hebei Provincial Key Laboratory of Intelligent Rehabilitation and Neuromodulation, Yanshan University, Qinhuangdao, China

**Keywords:** stair walking, knee biomechanics, muscle force analysis, OpenSim simulation, public health implications

## Abstract

**Objective:**

Stair ambulation is an essential daily activity associated with maintaining mobility and musculoskeletal health, and its biomechanical characteristics may influence fall risk. This study aimed to examine how different stair-walking strategies—single-step and double-step ascent—affect peri-knee muscle forces and joint loading using OpenSim-based musculoskeletal simulations, thereby providing biomechanical insights for public health–oriented interventions in injury prevention and rehabilitation.

**Methods:**

Synchronized kinematic and kinetic data were imported into OpenSim musculoskeletal models to compute knee joint angles, joint reaction forces, and peri-knee muscle forces. The simulated muscle activation levels were then compared with synchronized surface electromyography (sEMG) signals to validate the reliability of the simulations.

**Results:**

Results indicated that the double-step stair ascent mode produced significantly greater step length (*p* = 0.001) and larger peak hip (*p* = 0.002) and knee (*p* = 0.015) flexion compared with the single-step mode. Continuous-time SPM analysis further revealed that knee flexion remained significantly higher in the double-step mode throughout most of the single support phase (*p* < 0.001), while no significant differences were observed in knee joint reaction forces (*p* > 0.05). Muscle force analysis showed that quadriceps muscle force, including the quadriceps components (vastus medialis/lateralis/intermedius and rectus femoris) was significantly greater in the double-step mode during the early stage of the single support phase (*p* < 0.001). For the hamstrings, only the biceps femoris long head demonstrated significant differences between modes (*p* < 0.001), whereas the biceps femoris short head, semimembranosus, semitendinosus, and gastrocnemius showed no significant differences (*p* > 0.05).

**Conclusion:**

Double-Step Mode increased step length, hip mobility, and quadriceps activation without significantly elevating knee joint forces, supporting knee stability under higher mechanical demands. From a public health perspective, stair walking may enhance lower-limb muscle engagement and potentially contribute to the maintenance of balance control and mobility, while Single-Step Mode may be safer for individuals with knee impairments by minimizing joint stress and supporting rehabilitation.

## 1 Introduction

Stair ambulation is an indispensable functional activity in daily life, and its biomechanical characteristics and knee-loading mechanisms have long attracted attention in both sports science and rehabilitation medicine (1–3). Compared with level walking, stair ambulation requires greater lower-limb muscle force and joint range of motion to overcome changes in gravitational potential energy (4). Specifically, knee-flexion angles and extensor moments during stair ambulation increase by approximately 50% relative to level walking, and patellofemoral joint stress peaks can reach two to four times those observed during level gait (5, 6), resulting in substantially higher knee loading. These increased mechanical demands make stair walking a sensitive indicator of lower-limb function and a key component of physical performance assessment in both clinical and public health contexts. Moreover, different stair-walking strategies (e.g., single-step vs. double-step ascent) alter foot-support phases and the trajectory of the body’s center of mass, which may evoke distinct activation patterns in peri-knee musculature, further modulating knee-joint loading. However, the biomechanical mechanisms underlying these mode-dependent changes remain incompletely understood.

From a public health perspective, stair ambulation plays a critical role in maintaining mobility independence, preventing falls, and promoting musculoskeletal health across the lifespan. Impairments in stair negotiation ability are associated with reduced physical activity, increased risk of musculoskeletal disorders, and decreased quality of life, particularly among individuals with obesity, knee osteoarthritis, or diminished muscle force (7–9). Therefore, understanding the biomechanical strategies that influence knee loading during stair walking can inform preventive interventions, promote active lifestyles, and optimize rehabilitation outcomes.

Although previous studies have described the kinematic features and surface electromyography (sEMG) patterns of stair ambulation, conventional experimental approaches are limited in their ability to precisely quantify deep-muscle forces and activation levels (10). With advances in computational simulation, the open-source OpenSim platform provides a powerful tool to address this limitation (11). By integrating subject-specific musculoskeletal modeling, inverse dynamics, and dynamic-optimization algorithms, OpenSim can simulate muscle function throughout movement and yield accurate estimates of individual muscle forces, enabling in-depth investigation of the relationship between muscle loading and human movement mechanics (12, 13). The simulation accuracy of OpenSim has been experimentally validated in high-intensity tasks such as squatting and jumping (13–18).

Building on this foundation, the present study employs OpenSim-based simulations to construct biomechanical models for different stair-walking patterns, focusing on muscle forces around the knee during the single-support phase. The study aims to elucidate how stair-walking strategies influence muscle-force distribution and to investigate the knee’s biomechanical adaptation under different ascent modes. Compared with previous studies that mainly focused on kinematics or surface EMG patterns, this study systematically examines muscle forces around the knee using OpenSim, providing a detailed assessment of muscle function that has not been fully addressed in earlier research. Previous studies have shown that larger step heights and increased knee flexion during stair ascent significantly elevate quadriceps demand (5, 6). Therefore, we hypothesize that the Double-Step Mode will increase quadriceps muscle force and knee joint loading compared with the Single-Step Mode. Ultimately, the findings are expected to provide theoretical guidance for public health–oriented strategies supporting injury prevention, rehabilitation, and functional mobility enhancement.

## 2 Subjects and methods

### 2.1 Subjects

This study recruited 20 healthy adult males as participants, with a mean age of 20.6 ± 0.24 years, mean height of 182.12 ± 2.35 cm, and mean body weight of 75.82 ± 1.80 kg. The sample size was determined *a priori* using G*Power based on a paired t-test design, with an effect size of dz. = 0.9, a significance level of *α* = 0.05, and a desired statistical power of 0.95, which indicated a minimum required sample size of 19 participants. Inclusion criteria were as follows: (1) no congenital lower limb joint deformities or acquired musculoskeletal disorders; (2) no history of lower limb joint injury within the past 6 months; (3) physical capability to complete the stair walking task. Exclusion criteria included: (1) a history of lower limb surgery; (2) use of medications affecting neuromuscular function within 12 months prior to testing; (3) presence of neuromuscular disorders, psychological conditions, or cardiovascular and cerebrovascular diseases; (4) presence of acute pain symptoms. This study protocol was reviewed and approved by the Ethics Committee of Qinhuangdao First Hospital (Approval No.: 2025 K-135-01). All participants voluntarily signed a written informed consent form after being fully informed of the experimental procedures and agreed to participate in the study. In addition, written informed consent for the publication of identifying images and information in an open-access publication was obtained from all participants. All methods were carried out in accordance with relevant guidelines and regulations.

### 2.2 Experimental equipment

In this study, a Qualisys three-dimensional motion capture system equipped with eight high-speed infrared cameras operating at 200 Hz was used to record movement trajectories. Simultaneously, four Kistler three-dimensional force plates embedded within the staircase platform were utilized to collect kinetic data at a sampling rate of 2000 Hz. Additionally, a Delsys wireless surface electromyography (sEMG) system was employed to record muscle activation signals, with a sampling frequency of 2000 Hz and an inter-electrode distance of 10 mm, which allows precise recording of localized muscle activity while minimizing signal crosstalk. These experimental sEMG signals were used solely for validating the simulated muscle activation patterns obtained from OpenSim. Data collection was synchronized with a custom-designed five-step standardized staircase (step height: 15 cm ± 0.5 mm, step depth: 29 cm ± 0.5 mm), constructed according to the GB50352-2005 building code.

### 2.3 Testing procedure

**Figure 1:**
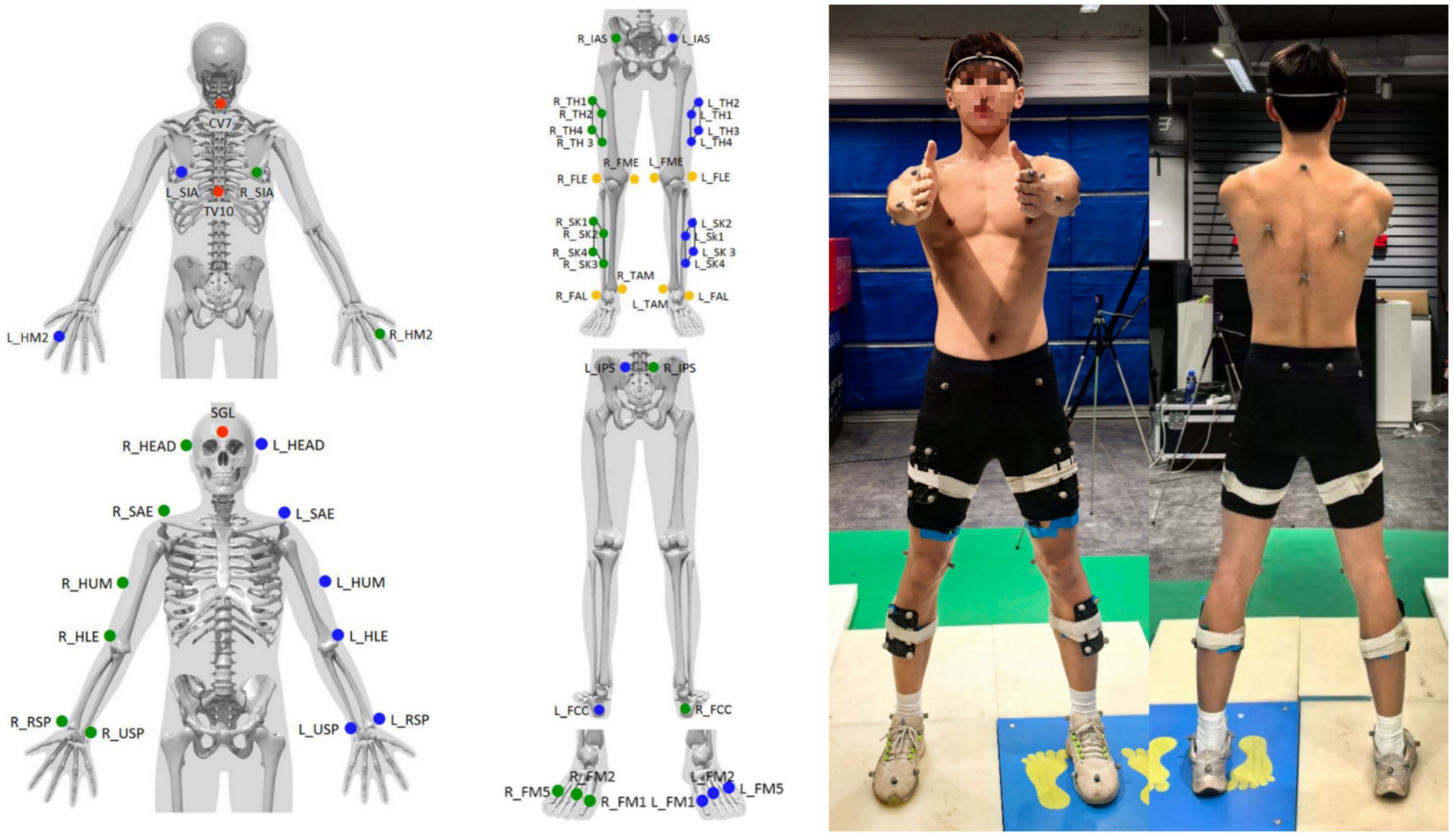
Schematic diagram of static data collection.

Participants first performed a standardized 5-min general warm-up, including 3 min of forward jogging, 1 min of lateral jogging, and 1 min of backward jogging (19). Reflective markers were then placed on anatomical landmarks across the body (Figure 1). Surface EMG electrodes were attached to target muscles of the right lower limb (Table 1) to validate the reliability of the OpenSim musculoskeletal model. Prior to electrode placement, the skin at each site was shaved if necessary and cleaned with alcohol to reduce impedance (<5 kΩ). Electrodes were placed along the muscle belly, parallel to the muscle fiber direction, following SENIAM guidelines (20). Subsequently, participants stood in the central area for a 10-s static calibration (Figure 1). Dynamic testing involved two stair ascent modes conducted in a randomized order, all participants stair ascent with their right leg: Single-Step Mode: Participants ascended the staircase step-by-step, with the supporting foot contacting only the adjacent upper step. Double-Step Mode: Participants ascended by skipping one step, with the supporting foot landing directly on the second upper step. Three valid trials were collected for each mode.

**Table 1:** Muscle identification and electrode placement.

Name	Electrode positions
Medial gastrocnemius	Over the maximal muscle bulge between the medial femoral epicondyle and the calcaneal tuberosity
Lateral gastrocnemius	Posterior to the medial femoral condyle, 1/3 along the line from the fibular head to the calcaneus
Rectus femoris	Midpoint of the line between the ASIS and the superior patellar pole
Tibialis anterior	At 25–33% of the leg length (from knee joint line to lateral malleolus), lateral to the tibial crest

### 2.4 OpenSim simulation workflow

**Figure 2:**
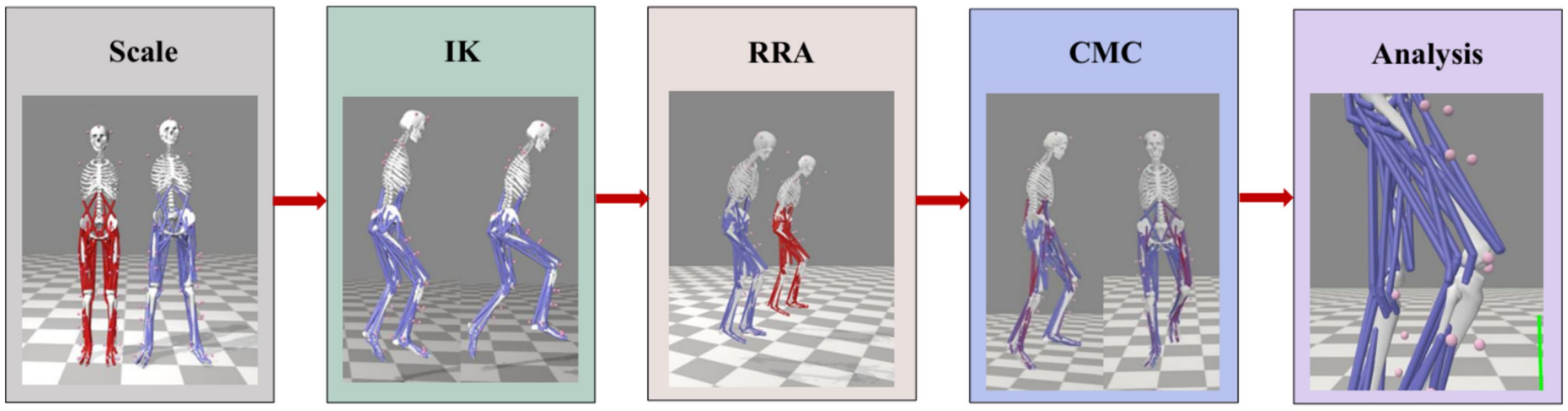
OpenSim simulation workflow.

The standard Gait2392 musculoskeletal model (23 degrees of freedom, 92 muscle-tendon units) was used for simulation modeling. Kinematic and kinetic data collected in C3D format were converted into .trc (trajectory files) and .mot (motion files) formats using MATLAB. The simulation workflow was as follows (see Figure 2): During the scaling phase (Scale), a subject-specific lower-limb musculoskeletal model was established by adjusting segment lengths, masses, and inertial properties based on each participant’s anthropometric data, using static calibration markers. A least squares optimization was applied to minimize the spatial coordinate errors between virtual model markers and experimental markers, ensuring that the root mean square error (RMSE) was controlled within 2 mm (21). Next, Inverse Kinematics (IK) was performed to calculate joint angles. To optimize dynamic consistency, the Residual Reduction Algorithm (RRA) was applied, with horizontal residual forces and torques constrained to within 5 and 0.5% of body weight, respectively, following standard OpenSim guidelines (22). Subsequently, the Computed Muscle Control (CMC) algorithm was used to estimate muscle activations and force distributions (23). Finally, the Analysis Tool module was utilized to calculate knee joint reaction forces (24).

### 2.5 Data processing

According to standard gait cycle segmentation criteria, the single support phase was defined as the period during which the tested leg (right lower limb) functioned as the stance limb, beginning at the contralateral toe-off and ending at the contralateral heel strike. Heel strike was identified as the instant when the vertical ground reaction force (vGRF) of the tested limb exceeded 5 N, whereas toe-off was identified as the instant when the vGRF dropped below 5 N (25). The single support phase was then normalized to 0–100% of the gait cycle. Knee joint reaction force and muscle force data were normalized to body mass. Raw EMG signals were processed with a 20–450 Hz band-pass filter, full-wave rectification, and a 10 Hz low-pass filter to obtain linear envelopes for subsequent analysis. The RMS method was then applied on the processed signals to quantify muscle activation levels. Finally, biomechanical parameters derived from the simulation were visualized using Origin software and smoothed with a cubic spline interpolation method.

### 2.6 Statistical analysis

Paired samples t-tests were conducted to examine the effects of stair-walking patterns on basic gait cycle characteristics and joint range of motion. Normality of the data was assessed using the Shapiro–Wilk test before performing paired t-tests. In addition, Statistical Parametric Mapping for paired samples (paired SPM{t}) was employed to evaluate the influence of different stair-walking patterns on knee flexion angles, knee joint reaction forces, and peri-knee muscle forces throughout the single support phase. All statistical analyses, including paired t-tests and paired SPM{t}, were performed using SPSS 20.0 (IBM Corp., USA) and MATLAB 2023a (MathWorks Inc., USA), respectively, with a significance threshold set at *p* < 0.05.

## 3 Research results

The primary outcome measures derived from OpenSim simulations included joint angles, muscle forces, joint reaction forces, and muscle activation levels during the single-support phase (Table 2).

**Table 2:** Outcome measures derived from OpenSim simulations.

Variable	Unit	Measurement phase	Description
Joint angle	°	Single-support phase	Hip flexion, Hip adduction, Hip external rotation, Knee flexion, and Ankle dorsiflexion angles
Muscle force	N/BW	Single-support phase	Vastus medialis, Vastus lateralis, Vastus intermedius, Rectus femoris, Biceps femoris (long and short heads), Semimembranosus, Semitendinosus, Medial and Lateral gastrocnemius
Joint reaction force	N/BW	Single-support phase	Knee joint reaction force
Muscle activation	%	Single-support phase	Simulated muscle activation levels obtained from OpenSim’s CMC algorithm

### 3.1 Model validation

**Figure 3:**
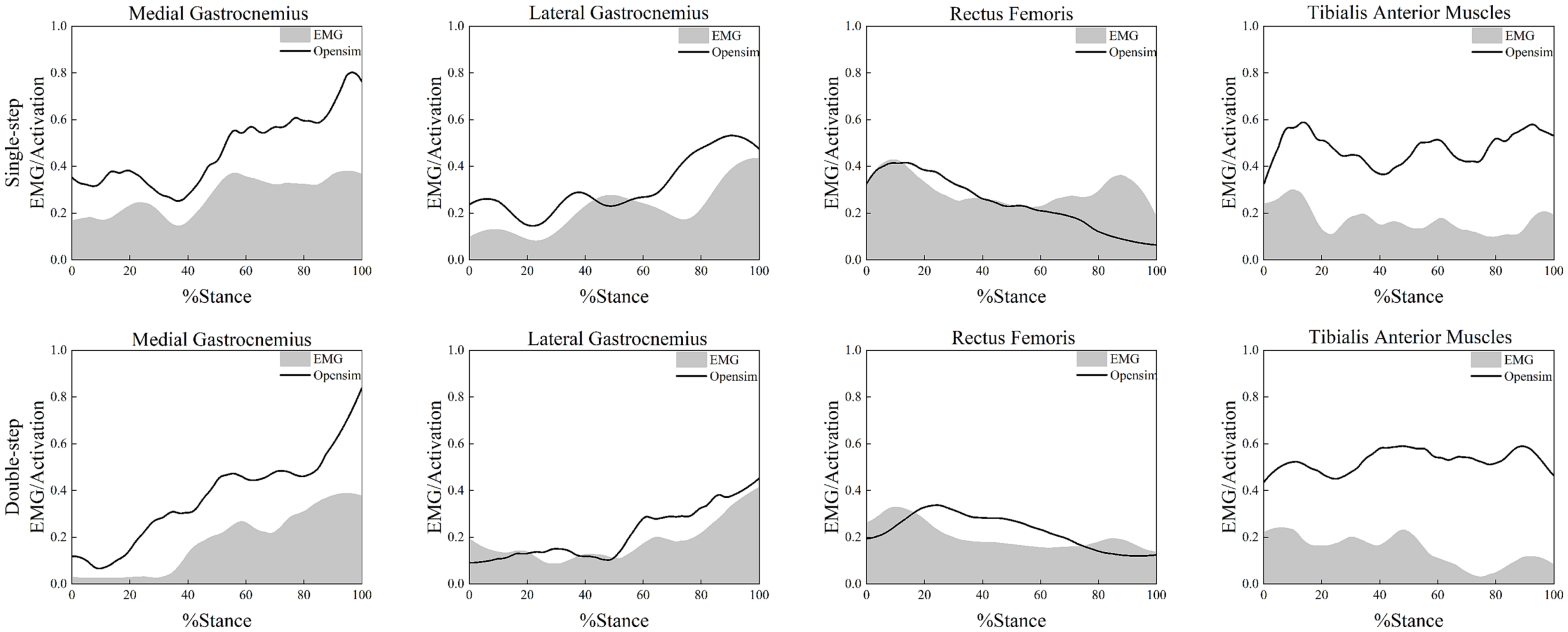
Comparison between EMG signals and muscle activation levels obtained by OpenSim optimization algorithms.

The root mean square (RMS) method was applied to process the collected sEMG signals, converting them into muscle activation indicators (ranging from 0 to 1, representing no activation to full activation, respectively). The processed results were compared with the muscle activation levels of the medial gastrocnemius, lateral gastrocnemius, rectus femoris, and tibialis anterior muscles calculated by the OpenSim optimization algorithms. The findings (see Figure 3) showed that during the single support phase, muscle activation patterns obtained from the OpenSim model were in good agreement with the experimentally collected surface EMG signals, confirming the high reliability of the constructed OpenSim model.

### 3.2 Kinematic characteristics of the gait cycle

Statistical analysis of the basic gait cycle characteristics (Table 3) revealed that the average step length in the double-step mode was significantly different from that in the single-step mode (*t* = −9.810, *p* = 0.001). Specifically, the average step length in the double-step mode was (1.39 ± 0.04 m) meters, which was significantly greater than that in the single-step mode (0.74 ± 0.11 m) meters.

**Table 3:** Basic characteristics of the gait cycle.

Parameter	Single-step mode	Double-step mode	*t*	*p*
Walking speed (m/s)	0.68 ± 0.08	0.72 ± 0.08	−0.754	0.477
Cadence (steps/min)	107.40 ± 7.53	107.4 ± 9.37	0.000	1.000
Average step length (m)	0.74 ± 0.11	1.39 ± 0.04	−9.810	0.001
Step width (m)	0.12 ± 0.02	0.15 ± 0.03	−1.504	0.207
Stance time (s)	0.15 ± 0.02	0.15 ± 0.03	0.173	0.871
Stance phase percentage (%)	63.20 ± 2.01	62.77 ± 1.28	−0.466	0.666
Swing phase percentage (%)	36.80 ± 2.01	37.22 ± 1.28	−0.466	0.666

Statistical analysis of joint range of motion during the single support phase under different stair ascent modes (Table 4) revealed significant differences in both hip and knee flexion between the single-step and double-step modes. Specifically, the hip flexion angle in the double-step mode (51.26 ± 8.34°) was significantly greater than that in the single-step mode (30.02 ± 5.94°, *t* = −6.816, *p* = 0.002). Similarly, the knee flexion angle was also significantly higher in the double-step mode (51.88 ± 9.87°) compared to the single-step mode (40.70 ± 5.63°, *t* = −4.080, *p* = 0.015).

**Table 4:** Range of motion of joints during the single support phase.

Parameter	Single-step mode	Double-step mode	*t*	*p*
Hip flexion (°)	30.02 ± 5.94	51.26 ± 8.34	−6.816	0.002
Hip adduction (°)	6.22 ± 3.15	6.46 ± 2.80	−0.143	0.893
Hip external rotation (°)	5.74 ± 2.80	8.64 ± 4.56	−1.262	0.296
Knee flexion (°)	40.70 ± 5.63	51.88 ± 9.87	−4.08	0.015
Ankle dorsiflexion (°)	11.70 ± 3.32	8.31 ± 4.37	2.436	0.072

### 3.3 Characteristics of knee joint angles and reaction forces

**Figure 4:**
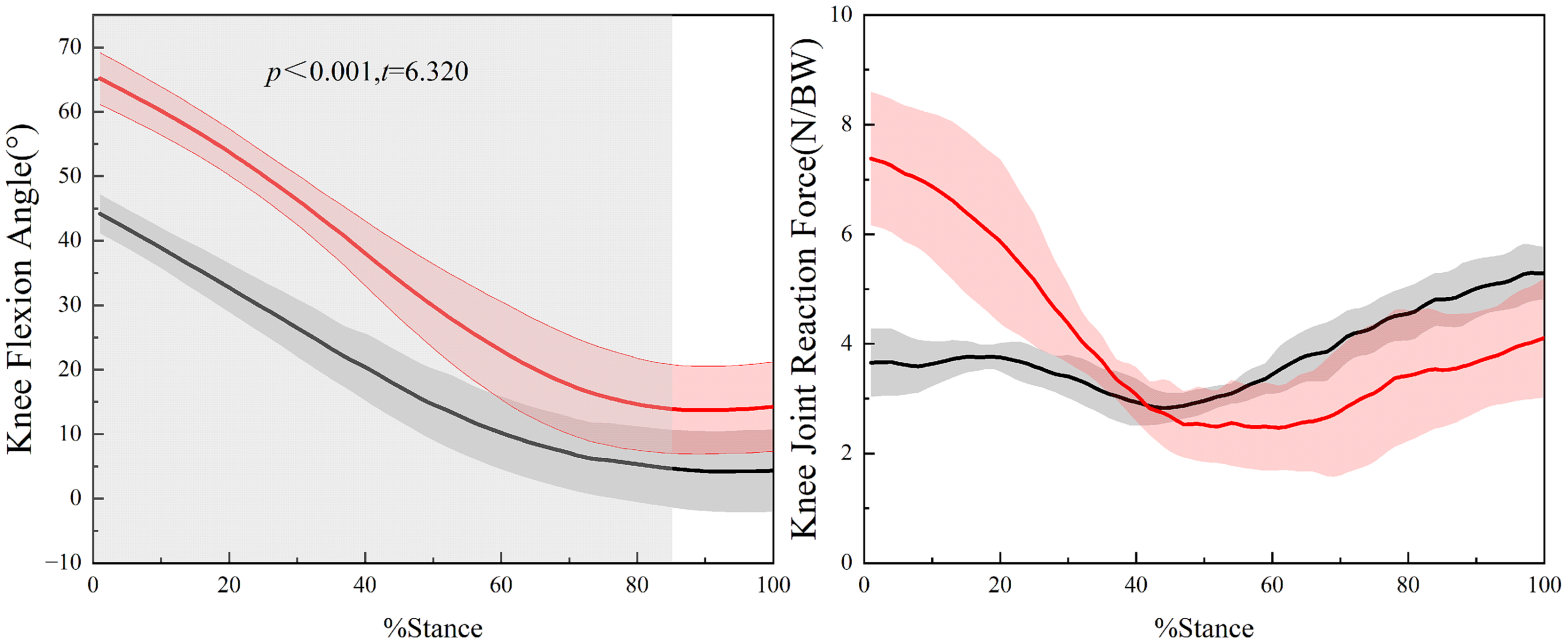
Comparison of knee flexion angle and knee joint reaction force under different stair walking modes. Black line represents the single-step mode, and red line represents the double-step mode.

Based on the continuous time analysis using SPM{t} (Figure 4), a statistically significant difference in knee flexion angle between the single-step and double-step modes was observed from the beginning of the single support phase to 85.75% of the gait cycle (*t* = 6.320, *p <* 0.001). However, no statistically significant differences in knee joint reaction forces were detected between the two stair walking modes across the entire gait cycle(*p >* 0.05).

### 3.4 Knee muscle force in different stair ascent modes

**Figure 5:**
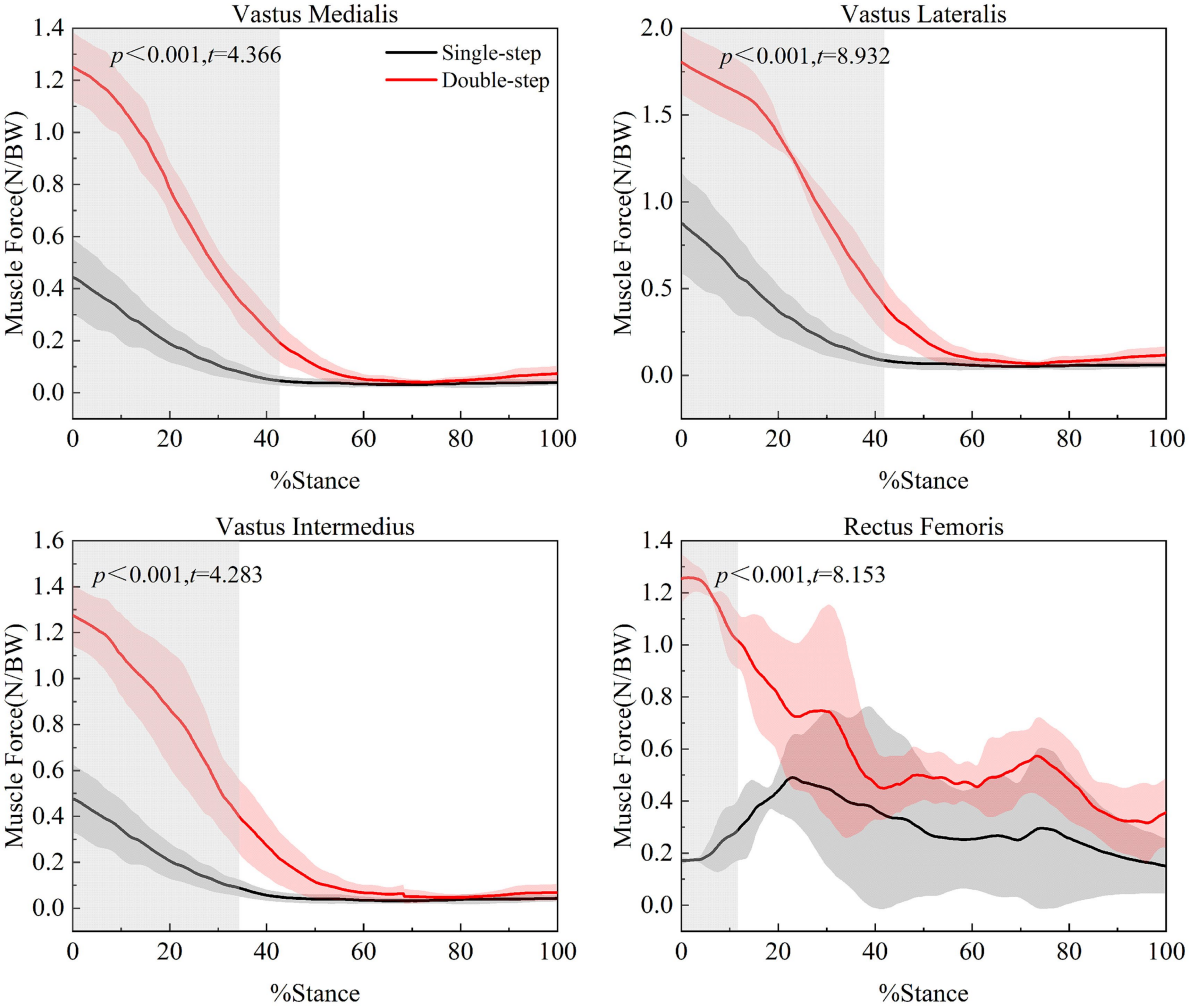
Muscle force curves of vastus medialis, vastus lateralis, vastus intermedius, and rectus femoris in different stair ascent modes. Black line represents the single-step mode, and red line represents the double-step mode.

Continuous-time analysis based on SPM{t} (Figure 5) revealed significant differences in the muscle force levels of various quadriceps muscles between the single-step and double-step modes. Specifically, significant intergroup differences were observed in: Vastus medialis from the beginning of the single support phase to 42.2% of the phase (*t* = 4.366, *p* < 0.001); Vastus lateralis up to 41.5% of the single support phase (*t* = 8.932, *p* < 0.001); Vastus intermedius from the beginning to 34.0% of the single support phase (*t* = 4.283, *p* < 0.001); Rectus femoris from the beginning to 11.2% of the single support phase (*t* = 8.153, *p* < 0.001).

**Figure 6:**
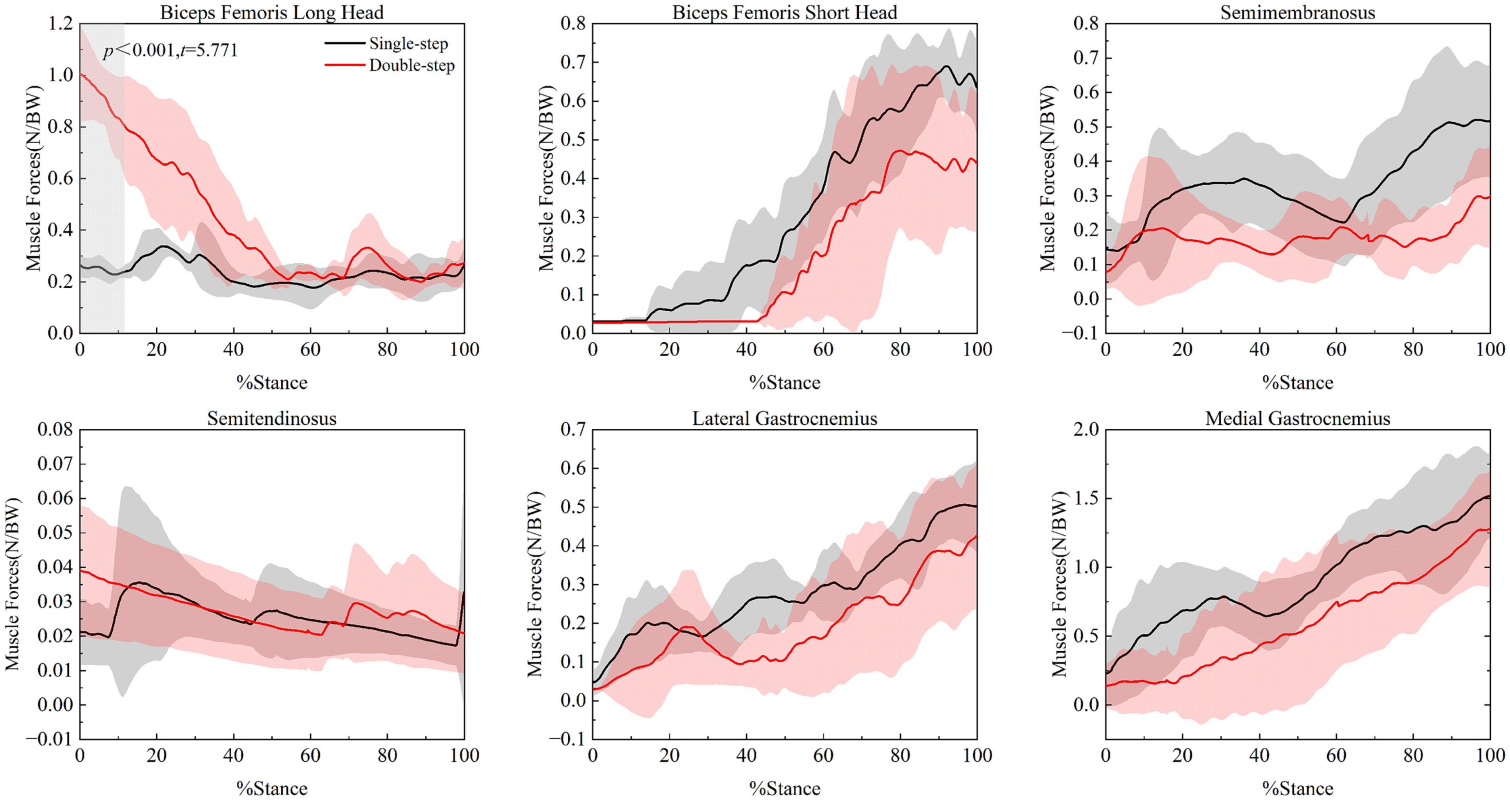
Muscle force curves of biceps femoris, semimembranosus, semitendinosus, and gastrocnemius in different stair ascent modes. Black line represents the single-step mode, and red line represents the double-step mode.

SPM{t} analysis results (Figure 6) show selective differences in the muscle force of the hamstrings between different stair ascent modes. Specifically, the biceps femoris long head exhibited significant muscle force differences between the single-step and double-step modes from the beginning of the single support phase to 11.6% of the phase (*t* = 5.771, *p* < 0.001). However, no significant intergroup differences were observed for the biceps femoris short head, semimembranosus, semitendinosus, or gastrocnemius (*p* > 0.05) in the statistical tests.

## 4 Discussion and analysis

This study, based on OpenSim simulation technology, aims to investigate the differential effects of single-step and double-step modes on gait cycle, kinematics, joint reaction forces, and muscle force characteristics during stair climbing. The primary focus is to reveal the biomechanical load differences in the knee joint across different stair ascent modes, providing biomechanical insights relevant to fall prevention, musculoskeletal health maintenance, and rehabilitation strategies. The results show significant differences between the single-step and double-step modes in terms of gait cycle step length, hip joint mobility, and muscle force characteristics.

The double-step mode requires a longer stride and greater hip and knee joint excursions compared to the single-step mode, reflecting higher mechanical demands and the need for coordinated lower limb joint control. This adaptation allows the body to overcome the vertical displacement of multiple steps simultaneously, necessitating increased hip and knee flexion and greater overall joint motion. Previous studies have demonstrated that step height, stride length, and obstacle characteristics influence lower limb joint angles and moments during stair ascent. For instance, Spanjaard et al. reported that step height and body mass significantly affect gastrocnemius fascicle behavior during stair climbing (26). Sakurai et al. observed that step-over strategies change with obstacle depth and height, reflecting age-related and task-specific adaptations (27). Hennah and Doumas found that dual-task or complex walking surfaces induce adaptive changes in step height, width, and stride length (28). More recent studies using advanced technologies confirm these effects: McCabe et al. reported that larger steps increase hip and knee excursions during stair ascent, and Chan et al. observed higher lower limb angular momentum and joint excursions in multi-step ascent (29, 30). Riener et al. also highlighted that stair inclination and step length modulate joint range of motion and mechanical requirements (31). In this study, the stair height and depth were 15 cm and 29 cm, respectively, which are slightly lower but comparable to those commonly used in previous studies, where stair height typically ranges from 17 to 21 cm and depth from 28 to 32 cm (32, 33). Hiemer et al. demonstrated that even small variations in stair dimensions can significantly affect knee kinematics and kinetics, indicating that step geometry plays a crucial role in shaping lower-limb motion strategies (33). Despite the relatively low stair height used in the present study, larger hip and knee joint excursions were observed, which may be attributed to the double-step mode that increases mechanical demands by requiring the body to overcome the vertical displacement of two steps simultaneously. This suggests that the observed kinematic differences are influenced not only by step geometry but also by the specific biomechanical requirements of the double-step mode. Together, these findings suggest that the double-step mode imposes greater biomechanical demands, requiring increased flexibility, stability, and control in the lower limb joints to ensure safe and efficient stair ascent. These adaptations not only reflect biomechanical efficiency but also offer valuable guidance for designing stair-climbing–based exercise or rehabilitation programs aimed at improving lower-limb muscle force and coordination.

Brach and Fujikawa’s studies pointed out that a larger knee flexion angle can improve mechanical leverage by elongating the quadriceps, thereby reducing quadriceps muscle force during stair climbing and enhancing the support capability of the lower limbs (34, 35). However, this study found that in the early phase of the single support, the knee flexion angle was significantly larger in the double-step mode, yet the quadriceps muscle force increased. This may be partially explained by the greater step length in the double-step mode, which could increase landing impact forces and consequently require higher quadriceps output to control and propel the body. Although eccentric and concentric mechanisms may be involved, this interpretation should be regarded as hypothetical since direct activation or joint moment data were not analyzed in this study (2, 3, 5). This phenomenon suggests that while a moderate increase in knee flexion angle may help enhance lower limb support, factors like step length variation and impact force absorption during stair climbing can increase the load on the quadriceps. Knee joint reaction forces did not differ significantly between the single-step and double-step modes. Nevertheless, a non-significant trend toward higher peak forces was observed in the double-step mode (7.38 BW vs. 5.29 BW in the single-step mode), which may be partially attributed to increased step length and quadriceps muscle force, leading to a forward shift of the center of mass and greater loading demands on the stance leg during the single-support phase (2, 3, 36). Comparatively, *in vivo* measurements of tibiofemoral contact forces using instrumented knee implants report peak resultant forces of approximately 3.16 BW during stair ascent and up to 3.46 BW during stair descent in five subjects (37, 38). While our simulation values exceed these in-vivo measurements, the relative increase observed in the double-step mode aligns with the trend reported in previous studies. The differences may stem from model assumptions, stair geometry, and participant loading conditions. Therefore, understanding the mechanical implications of different stair-climbing modes can inform the development of tailored exercise prescriptions that balance training benefits with joint protection, particularly for older adults or individuals with knee disorders.

Additionally, in the double-step mode, the muscle forces of the quadriceps components—including vastus medialis, vastus lateralis, vastus intermedius, and rectus femoris—significantly increased during the early phase of the single-support phase. This suggests that the quadriceps exert greater eccentric control to counteract larger impact forces and maintain knee stability (39, 40). Notably, the vastus medialis showed a marked increase from the beginning to 42.2% of the single-support phase, likely related to higher external rotation torque caused by the longer step length. As the vastus medialis primarily prevents lateral patellar displacement, its increased activation may help maintain knee joint stability (41). Overall, the activation levels of quadriceps components vary according to gait characteristics and stair-walking mode.

Similarly, significant differences in muscle force were observed in the vastus lateralis from the beginning of the single-support phase to the 41.5% phase. This may be due to the increased activation of the vastus medialis in response to the external rotation torque, which leads to a greater need for cooperation from the vastus lateralis. As a result, the vastus lateralis must achieve higher activation levels to maintain knee joint symmetry and balance (42). This finding is partly consistent with the results of Akima et al. (43), who used functional MRI to analyze motor unit recruitment and neuromuscular metabolism during isokinetic concentric knee extension. While their study involved controlled, single-joint contractions, the present research examines dynamic stair-climbing conditions. Nevertheless, their observation that the vastus medialis and vastus lateralis are activated synergistically may provide insight into the coordination of quadriceps components, helping to maintain patellar stability during complex lower-limb movements (40, 44).

Additionally, significant differences were observed in the vastus intermedius from the beginning of the single-support phase to the 34.0% phase. This muscle, located on the front of the femur, primarily functions in knee extension. In the double-step mode, due to the increased knee flexion angle, the vastus intermedius needs to exert more force to complete a greater range of extension. Notably, significant differences in the rectus femoris only appeared from the beginning of the single-support phase to the 11.2% phase. As the rectus femoris is a bi-joint muscle, it also bears additional hip flexion load in the double-step mode, resulting in a higher muscle force requirement during the initial phase of the single-support phase. This emphasizes the importance of maintaining balanced quadriceps muscle force and neuromuscular coordination in stair-related movements to reduce the risk of patellofemoral pain or instability.

In contrast to the significant differences observed in the quadriceps, the selective activation of the hamstring muscles was more limited. Only the long head of the biceps femoris exhibited significant inter-group differences during the early phase of the single-support phase. The long head of the biceps femoris, a bi-joint muscle, is involved in both knee flexion and hip extension. In the double-step mode, the increased step length necessitates a larger hip extension angle, which increases the activation demand of the long head of the biceps femoris. However, the short head of the biceps femoris, semimembranosus, semitendinosus, and gastrocnemius did not show significant inter-group differences in statistical tests. This may be because these muscles primarily contribute to knee joint stability during the single-support phase by controlling flexion and rotation, rather than providing primary power output, as supported by previous studies on hamstring and gastrocnemius function (4, 45). In contrast, the quadriceps muscles play a more dominant role during this phase, with their synergistic activation contributing to both knee stability and power regulation. These findings underscore the need to incorporate both quadriceps and hamstring coactivation strategies in functional training to enhance knee stability and reduce overuse injuries.

In summary, in the double-step mode, the foot contact point is higher, and the impact force is greater. The quadriceps must generate greater muscle force, which likely helps support knee stability and attenuate joint loading. In contrast, hamstring muscle forces did not differ significantly between the two stair descent modes during the single-support phase.

From a public health perspective, these biomechanical insights may provide theoretical support for developing stair-based exercise strategies aimed at enhancing lower-limb muscle function and promoting safe mobility. For healthy individuals or athletes, incorporating Double-Step Mode may elicit distinct activation patterns in the quadriceps and hamstrings, which could have implications for dynamic knee stability. However, for individuals with knee joint injury or degeneration, single-step mode may help reduce joint loading, minimize muscle fatigue, and lower injury risk. Overall, tailoring stair-climbing patterns to individual physical conditions may help support long-term musculoskeletal health.

Several limitations should be acknowledged in this study. First, the sample was homogeneous, consisting only of healthy young males, which may limit the generalizability of the findings to females, older adults, or clinical populations. Second, the musculoskeletal simulations were based on the generic Gait2392 model, which has simplified knee mechanics and a limited number of muscles; while this model is widely used and reliable for general musculoskeletal simulations, higher-fidelity models may provide additional insights in future studies. Third, the validation of simulated muscle forces using surface EMG was limited to four muscles—the rectus femoris, tibialis anterior, medial gastrocnemius, and lateral gastrocnemius. Although the hamstrings were not directly measured, changes in the measured muscles partially reflect the functional patterns of major knee-joint muscles. Finally, while OpenSim provides detailed estimates of muscle forces, such simulations require motion capture systems, force platforms, and modeling expertise that may not be readily available in typical clinical settings; more clinically accessible tools (e.g., portable EMG or wearable sensors) could be explored as potential alternatives for evaluating knee muscle function in routine practice.

Building on the current findings, future studies could prioritize stair descent analysis and investigation of clinical populations with knee disorders. Stair descent imposes unique biomechanical challenges compared to ascent, including increased eccentric loading of peri-knee muscles, higher joint reaction forces during early stance, and greater demands on dynamic balance to maintain stability. Examining these factors in healthy adults would provide fundamental insights into knee joint control strategies under higher-demand conditions. Extending this research to clinical populations, such as individuals with osteoarthritis or post-surgical knee recovery, could elucidate how pathological adaptations affect muscle activation patterns, joint loading, and stability during stair locomotion. Understanding these adaptations is crucial for designing evidence-based rehabilitation programs and targeted exercise interventions aimed at minimizing knee joint stress and preventing falls.

## 5 Conclusion

This study, using OpenSim simulation, examined the effects of single-step mode and double-step mode of stair walking on gait kinematics, joint reaction forces, and peri-knee muscle force. The double-step mode increased step length and hip mobility and induced greater quadriceps muscle force, while knee joint reaction forces showed no significant difference between modes. These findings suggest that the double-step mode imposes higher muscular demands for movement control, which may contribute to maintaining knee stability rather than indicating increased joint loading. From a public health perspective, stair walking may serve as a beneficial functional activity to maintain lower-limb strength and support balance ability, single-step mode may be more suitable for individuals with knee impairments to reduce joint stress and support safe rehabilitation.

## Data Availability

The raw data supporting the conclusions of this article will be made available by the authors, without undue reservation.

## References

[1] Sliepen M Mauricio E Lipperts M Grimm B Rosenbaum D. Objective assessment of physical activity and sedentary behaviour in knee osteoarthritis patients–beyond daily steps and total sedentary time. BMC Musculoskelet Disord. (2018) 19:1–10. doi: 10.1186/s12891-018-1980-3, PMID: 29471878 PMC5824451

[2] Protopapadaki A Drechsler WI Cramp MC Coutts FJ Scott OM. Hip, knee, ankle kinematics and kinetics during stair ascent and descent in healthy young individuals. Clin Biomech. (2007) 22:203–10. doi: 10.1016/j.clinbiomech.2006.09.010, PMID: 17126461

[3] McFadyen BJ Winter DA. An integrated biomechanical analysis of normal stair ascent and descent. J Biomech. (1988) 21:733–44. doi: 10.1016/0021-9290(88)90282-5, PMID: 3182877

[4] Grimmer M Zeiss J Weigand F Zhao G. Joint power, joint work and lower limb muscle activity for transitions between level walking and stair ambulation at three inclinations. PLoS One. (2023) 18:e0294161. doi: 10.1371/journal.pone.0294161, PMID: 37972031 PMC10653464

[5] Nadeau S McFadyen BJ Malouin F. Frontal and sagittal plane analyses of the stair climbing task in healthy adults aged over 40 years: what are the challenges compared to level walking? Clin Biomech. (2003) 18:950–9. doi: 10.1016/S0268-0033(03)00179-7, PMID: 14580839

[6] Brechter H Powers CM. Patellofemoral stress during walking in persons with and without patellofemoral pain. Med Sci Sports Exerc. (2002) 34:1582–93. doi: 10.1097/00005768-200210000-00009 12370559

[7] Menoth Mohan D Al Anouti F Kohli N Khalaf K. Association of obesity with musculoskeletal health and functional mobility in females – a systematic review. Int J Obes. (2025) 49:2184–205. doi: 10.1038/s41366-025-01881-8, PMID: 40968131 PMC12583141

[8] Rice PE Pate GA Hill RD DeVita P Messier SP. The association between obesity, knee pain, and gait during stair descent in older adults with knee osteoarthritis. Clin Biomech. (2024) 114:106228. doi: 10.1016/j.clinbiomech.2024.106228, PMID: 38518651

[9] Leutzinger TJ Kingston DC Dinkel DM Wellsandt E Knarr BA. Differences in knee joint moments between individuals who are living with obesity and those of a healthy weight when negotiating stairs. Knee. (2024) 49:217–25. doi: 10.1016/j.knee.2024.07.006, PMID: 39043017 PMC12376230

[10] Li X Chen J Li X Yu Z. Liu H. Li S. Temporal analysis method of lower limb muscle activation based on OpenSim[C]//Chinese intelligent systems conference. Singapore: Springer Nature Singapore, (2024). p. 556–568.

[11] Seth A Hicks JL Uchida TK Habib A Dembia CL Dunne JJ . OpenSim: simulating musculoskeletal dynamics and neuromuscular control to study human and animal movement. PLoS Comput Biol. (2018) 14:e1006223. doi: 10.1371/journal.pcbi.1006223, PMID: 30048444 PMC6061994

[12] Rasmussen J Damsgaard M Voigt M. Muscle recruitment by the min/max criterion—a comparative numerical study. J Biomech. (2001) 34:409–15. doi: 10.1016/S0021-9290(00)00191-3, PMID: 11182135

[13] Roelker SA Caruthers EJ Hall RK Pelz NC Chaudhari AMW Siston RA. Effects of optimization technique on simulated muscle activations and forces. J Appl Biomech. (2020) 36:259–78. doi: 10.1123/jab.2018-0332, PMID: 32663800

[14] Jessup LN Kelly LA Cresswell AG Lichtwark GA. Validation of a musculoskeletal model for simulating muscle mechanics and energetics during diverse human hopping tasks. R Soc Open Sci. (2023) 10:230393. doi: 10.1098/rsos.230393, PMID: 37885982 PMC10598413

[15] Lu Y Mei Q Peng HT Li J Wei C Gu Y. A comparative study on loadings of the lower extremity during deep squat in Asian and Caucasian individuals via OpenSim musculoskeletal modelling. Biomed Res Int. (2020) 2020:7531719. doi: 10.1155/2020/7531719

[16] Kothurkar R Lekurwale R Gad M Rathod CM. Estimation and comparison of knee joint contact forces during heel contact and heel rise deep squatting. Indian J Orthop. (2023) 57:310–8. doi: 10.1007/s43465-022-00798-y, PMID: 36777124 PMC9880086

[17] Hosseini Nasab SH Smith CR Maas A Vollenweider A Dymke J Schütz P . Uncertainty in muscle–tendon parameters can greatly influence the accuracy of knee contact force estimates of musculoskeletal models. Front Bioeng Biotechnol. (2022) 10:808027. doi: 10.3389/fbioe.2022.808027, PMID: 35721846 PMC9204520

[18] Imani Nejad Z Khalili K Hosseini Nasab SH Schütz P Damm P Trepczynski A . The capacity of generic musculoskeletal simulations to predict knee joint loading using the CAMS-knee datasets. Ann Biomed Eng. (2020) 48:1430–40. doi: 10.1007/s10439-020-02465-5, PMID: 32002734 PMC7089909

[19] Chaouachi A Castagna C Chtara M Brughelli M Turki O Galy O . Effect of warm-ups involving static or dynamic stretching on agility, sprinting, and jumping performance in trained individuals. J Strength Cond Res. (2010) 24:2001–11. doi: 10.1519/JSC.0b013e3181aeb181, PMID: 19855310

[20] Hermens HJ Freriks B Disselhorst-Klug C Rau G. Development of recommendations for SEMG sensors and sensor placement procedures. J Electromyogr Kinesiol. (2000) 10:361–74. doi: 10.1016/S1050-6411(00)00027-4, PMID: 11018445

[21] Delp SL Anderson FC Arnold AS Loan P Habib A John CT . OpenSim: open-source software to create and analyze dynamic simulations of movement. IEEE Trans Biomed Eng. (2007) 54:1940–50. doi: 10.1109/TBME.2007.901024, PMID: 18018689

[22] Akbas T Neptune RR Sulzer J. Neuromusculoskeletal simulation reveals abnormal rectus femoris-gluteus medius coupling in post-stroke gait. Front Neurol. (2019) 10:301. doi: 10.3389/fneur.2019.00301, PMID: 31001189 PMC6454148

[23] Thelen DG Anderson FC. Using computed muscle control to generate forward dynamic simulations of human walking from experimental data. J Biomech. (2006) 39:1107–15. doi: 10.1016/j.jbiomech.2005.02.010, PMID: 16023125

[24] Walter JP Pandy MG. Dynamic simulation of knee-joint loading during gait using force-feedback control and surrogate contact modelling. Med Eng Phys. (2017) 48:196–205. doi: 10.1016/j.medengphy.2017.06.043, PMID: 28712529

[25] Leitch J Stebbins J Paolini G Zavatsky AB. Identifying gait events without a force plate during running: a comparison of methods. Gait Posture. (2011) 33:130–2. doi: 10.1016/j.gaitpost.2010.06.009, PMID: 21084195

[26] Spanjaard M Reeves ND Van Dieen JH Baltzopoulos V Maganaris CN. Influence of step-height and body mass on gastrocnemius muscle fascicle behavior during stair ascent. J Biomech. (2008) 41:937–44. doi: 10.1016/j.jbiomech.2008.01.003, PMID: 18282576

[27] Sakurai R Miura Y Kodama K. Effect of obstacle depth and height on step-over behavior: focus on age-related changes. Hum Mov Sci. (2025) 99:103323. doi: 10.1016/j.humov.2025.103323, PMID: 39854824

[28] Hennah C Doumas M. Dual-task walking on real-world surfaces: adaptive changes in walking speed, step width and step height in young and older adults. Exp Gerontol. (2023) 177:112200. doi: 10.1016/j.exger.2023.112200, PMID: 37160198

[29] McCabe MV Van Citters DW Chapman RM. Hip joint angles and moments during stair ascent using neural networks and wearable sensors. Bioengineering. (2023) 10:784. doi: 10.3390/bioengineering10070784, PMID: 37508811 PMC10376156

[30] Chan DOM Subasinghe Arachchige RSS Wang S Chan PPK Cheung RTH. Whole-body angular momentum during stair ascent and descent in individuals with and without knee osteoarthritis. Sci Rep. (2024) 14:30754. doi: 10.1038/s41598-024-80423-0, PMID: 39730473 PMC11681112

[31] Riener R Rabuffetti M Frigo C. Stair ascent and descent at different inclinations. Gait Posture. (2002) 15:32–44. doi: 10.1016/S0966-6362(01)00162-X, PMID: 11809579

[32] Makani A Shirazi-Adl SA Ghezelbash F. Computational biomechanics of human knee joint in stair ascent: muscle-ligament-contact forces and comparison with level walking. Int. J. Numerical Methods Biomed. Eng. (2022) 38:e3646. doi: 10.1002/cnm.3646, PMID: 36054682

[33] Trinler UK Baty F Mündermann A Fenner V Behrend H Jost B . Stair dimension affects knee kinematics and kinetics in patients with good outcome after TKA similarly as in healthy subjects. J Orthop Res. (2016) 34:1753–61. doi: 10.1002/jor.23181, PMID: 26844935

[34] Brach JS Berthold R Craik R VanSwearingen JM Newman AB. Gait variability in community-dwelling older adults. J Am Geriatr Soc. (2001) 49:1646–50. doi: 10.1046/j.1532-5415.2001.t01-1-49274.x, PMID: 11843998

[35] Fujikawa K Seedhom BB Wright V. Biomechanics of the patello-femoral joint. Part I: a study of the contact and the congruity of the patello-femoral compartment and movement of the patella. Eng Med. (1983) 12:3–11. doi: 10.1243/EMED_JOUR_1983_012_004_02, PMID: 6682059

[36] Adamczyk PG Kuo AD. Redirection of center-of-mass velocity during the step-to-step transition of human walking. J Exp Biol. (2009) 212:2668–78. doi: 10.1242/jeb.027581, PMID: 19648412 PMC2726857

[37] Kutzner I Heinlein B Graichen F Bender A Rohlmann A Halder A . Loading of the knee joint during activities of daily living measured in vivo in five subjects. J Biomech. (2010) 43:2164–73. doi: 10.1016/j.jbiomech.2010.03.046, PMID: 20537336

[38] Fregly BJ Besier TF Lloyd DG Delp SL Banks SA Pandy MG . Grand challenge competition to predict in vivo knee loads. J Orthop Res. (2012) 30:503–13. doi: 10.1002/jor.22023, PMID: 22161745 PMC4067494

[39] Lewek MD Rudolph KS Snyder-Mackler L. Quadriceps femoris muscle weakness and activation failure in patients with symptomatic knee osteoarthritis. J Orthop Res. (2004) 22:110–5. doi: 10.1016/S0736-0266(03)00154-2, PMID: 14656668 PMC3073134

[40] Bolgla LA Uhl TL. Electromyographic analysis of hip rehabilitation exercises in a group of healthy subjects. J Orthop Sports Phys Ther. (2005) 35:487–94. doi: 10.2519/jospt.2005.35.8.487, PMID: 16187509

[41] Tanino Y Yoshida T Yamazaki W Fukumoto Y Nakao T Suzuki T. Function of the distal part of the vastus medialis muscle as a generator of knee extension twitch torque. J. Funct. Morphol. Kinesiol. (2020) 5:98. doi: 10.3390/jfmk5040098, PMID: 33467313 PMC7804882

[42] Shu L Yang X He H Chen B Chen L Ni Q. Morphological study of the vastus medialis oblique in recurrent patellar dislocation based on magnetic resonance images. BMC Med Imaging. (2021) 21:1–10. doi: 10.1186/s12880-020-00542-8, PMID: 33407236 PMC7788929

[43] Çinarli FS Çinarli S Kafkas E. Comparison of thigh muscle activations in single leg exercises: bench squat, step-up, airborne lunge. Pedagogy Phys. Cult. Sports. (2021) 25:342–8. doi: 10.15561/26649837.2021.0601

[44] Akima H Takahashi H Kuno S Katsuta S. Coactivation pattern in human quadriceps during isokinetic knee-extension by muscle functional MRI. Eur J Appl Physiol. (2004) 91:7–14. doi: 10.1007/s00421-003-0942-z, PMID: 14551776

[45] Frigo CA Grossi M Donno L. Loads on the knee joint ligaments during stair climbing. Appl Sci. (2023) 13:7388. doi: 10.3390/app13137388

